# Erythrocyte glycocalyx sensitivity to sodium is associated with salt sensitivity of blood pressure in women but not men

**DOI:** 10.3389/fnut.2024.1334853

**Published:** 2024-03-08

**Authors:** Sepiso K. Masenga, Benson M. Hamooya, Kaushik P. Patel, Annet Kirabo

**Affiliations:** ^1^HAND Research Group, School of Medicine and Health Sciences, Mulungushi University, Livingstone, Zambia; ^2^Department of Medicine, Vanderbilt University Medical Center, Nashville, TN, United States; ^3^Department of Cellular and Integrative Physiology, University of Nebraska Medical Center, Omaha, NE, United States; ^4^Vanderbilt Center for Immunobiology, Vanderbilt University Medical Center, Nashville, TN, United States; ^5^Vanderbilt Institute for Infection, Immunology and Inflammation, Vanderbilt University Medical Center, Nashville, TN, United States; ^6^Vanderbilt Institute for Global Health, Vanderbilt University Medical Center, Nashville, TN, United States

**Keywords:** erythrocyte glycocalyx sensitivity to sodium, salt sensitivity of blood pressure, immediate pressor response to dietary salt, blood pressure, systolic blood pressure, diastolic blood pressure, sodium chloride, salt

## Abstract

**Background:**

While salt sensitivity of blood pressure (SSBP) is a risk factor for hypertension, end-organ damage and death, most studies are conducted in western countries and in White people. We previously found that the prevalence of SSBP in Blacks living in Sub-Saharan Africa is as high as 75–80% like what has been reported in the west. Erythrocyte glycocalyx sensitivity to sodium (eGCSS), a marker of sodium-induced damage to the erythrocyte and vascular endothelial glycocalyx is thought to be related to blood pressure perturbations associated with salt intake. We hypothesized that SSBP correlates with eGCSS differently in men and women in Black people.

**Methods:**

We conducted a cross sectional study using data from our recent clinical trial from Livingstone University Teaching Hospital among 117 normotensive young adults. We used a “salt blood test” to determine eGCSS and an immediate pressor response to oral salt (IPROS) for the diagnosis of SSBP.

**Results:**

The proportion of males were equal to females and the median age (interquartile range) of the participants was 29 (22–45) years. The eGCSS scores were higher in salt-resistant females compared to salt-sensitive females and males. eGCSS correlated negatively with SSBP (AOR 0.98, 95% CI 0.97–0.99, *p* = 0.008), however, this relationship was driven by female sex and abrogated by male sex. Although blood pressure elevations exhibited a sustained bimodal pattern in both sexes, in males, systolic and diastolic blood pressure never returned to baseline during the time course as it did in females.

**Conclusion:**

In this study, eGCSS correlated negatively with SSBP in black women but not in black men and the pressor response to dietary salt was significantly higher in men compared to women. These results suggest that women tend to have a higher disruption of the vascular endothelial glycocalyx by an acute salt load, implying that acute changes in blood pressure may not be driven directly by the endothelial glycocalyx. Our findings suggest a novel mechanism linking eGCSS and SSBP with potential implications for sex differences in salt-induced cardiovascular disease.

**Clinical trial registration**: https://clinicaltrials.gov/, identifier [NCT04844255].

## 1 Introduction

Excess dietary salt is associated with future development of hypertension, cardiovascular disease and higher mortality (1–3). Although the pressor effect of long term excess in salt intake has implications for a chronic blood pressure elevation, we have previously demonstrated an immediate pressor response to oral dietary salt (IPROS) in salt sensitivity of blood pressure (SSBP), in a young population of normotensive individuals (4). Previously, it has been suggested that the contribution of excess dietary salt to hypertension is mediated by salt’s direct damaging effect on the endothelial and erythrocyte glycocalyx resulting in the extravasation of fluids and sodium, inflammation and hypertension (5). We have reported recently that high salt intake was associated with poor vascular sodium buffering capacity or high erythrocyte glycocalyx sensitivity to sodium (eGCSS) in a cohort of Black people in Zambia (6). Previous studies have reported sex differences in blood pressure regulation (7–10) including SSBP (11). Although SSBP is prevalent in both women and men, there is more evidence showing that premenopausal women have higher prevalence compared to men and menopause increases this prevalence and severity (12–15). However, the role of eGCSS in sex-specific responses to an acute salt-load or SSBP remains to be known. Moreover, most studies on SSBP and eGCSS were conducted in White populations whereas Black populations, particularly in Sub-Sahara Africa have not been studied. Thus, the goal of this study was to determine the sex differences in the relationship between eGCSS and SSBP in a young black population from sub-Saharan Africa (SSA).

## 2 Materials and methods

### 2.1 Study design and setting

This was a cross sectional study, where we used secondary data from a time series clinical trial conducted at Livingstone University Teaching Hospital among a young normotensive black population (4).

### 2.2 Study procedure and recruitment

The recruitment, study procedures and demographic characteristics of this population have been described in detail previously (4). Briefly, we selected only participants from the dataset of 127 participants (4) who had complete information on all the variables (employment status, marital status, HIV status, ankle brachial index) including eGCSS (*n* = 117). A total of 117 participants were studied who had undergone a salt loading procedure to diagnose SSBP based on an IPROS (4) after an overnight fast of at least 8 hrs. Participants rested for 40 min prior to receiving 2 g of oral salt (~788 mg or 34 mmol of sodium) and blood pressure was monitored and recorded for 120 min in 10-min intervals.

Blood pressure was measured using an automated validated sphygmomanometer (Omron HEM-7120; Omron Healthcare Co., Limited, Kyoto, Japan) while adhering to accurate blood pressure monitoring procedures (4). Sociodemographic characteristics were collected prior to the intervention. The ankle-brachial index was recorded with the aid of a Sonotrax Vascular Doppler (Ultrasonic pocket doppler; Shanghai International Holding Corp. GmbH, Hamburg, Germany), a vascular probe and a sphygmomanometer with attached gauge.

The eGCSS determination was described in detail by Oberleithner and Wilhelmi (16) and we recently described it (6), as well as others (17). Briefly, 50 μL of capillary blood is used to determine erythrocyte sedimentation for 60 min in a hematocrit tube. eGCSS was calculated as a percentage relative to the standard values of 21.4 and 26.1 mm for males and females, respectively (16).

### 2.3 Definitions and outcomes

We diagnosed SSBP based on our previous clinical trial study described in detail (4). Briefly, SSBP was diagnosed as mean arterial pressure (MAP) difference of ≥10 mmHg between the baseline and the highest MAP during the 120-min time course after salt loading. To compute the *ankle-brachial* index, we used the higher of two systolic blood pressure (SBP) from the dorsalis pedis and posterior tibial arteries in the foot and divided by the higher brachial SBP in the participant’s arms. The measurements were taken with the participant in supine position.

### 2.4 Data analysis

We used IBM SPSS ver. 22.0 (IBM Corp., Armonk, NY, United States) and GraphPad Prism version 9.5.1 for all descriptive and inferential statistical analyses and graphs. We used Mann–Whitney *U* test to compare age, body mass index, ankle brachial index, fasting blood sugar, eGCSS and red blood cell count between salt sensitive and salt resistant individuals. To compare systolic and diastolic means between males and females, we used ANOVA, Welch’s test with multiple comparisons. To compare distributions between categorical variables and SSBP, we used the Chi-square test. To determine correlates of SSBP we used univariable and multivariable logistic regression models. Age, sex and body mass index have been reported in multiple studies to influence blood pressure responses to salt intake (14, 18–21). We therefore included these three variables in the multiple logistic regression model to control for confounding.

### 2.5 Ethical approval

We received ethics clearance and approval from the University of Zambia Biomedical Research Ethics committee (IRB00001131 of IORG0000774) and the National Health Research Ethics Board under reference number 981–2020. All participants signed an Institutional Review Board-approved consent form after a thorough explanation of the protocol before being included in the study. The study was conducted in accordance with the Declaration of Helsinki.

## 3 Results

### 3.1 Study characteristics

The median age (interquartile range, IQR) of participants was 29 years (22–45 years) with 1:1 male-to-female ratio (Supplementary Table S1). Most participants were single (55.6%), unemployed (74.4%), HIV negative (96%), salt sensitive (61.5%), and had high eGCSS (64.1%) with a normal ankle-brachial index (94.9%), red blood cell count, fasting blood sugar and body mass index. Apart from eGCSS, all variables were comparable between salt sensitive and salt resistant groups.

### 3.2 Sex differences in blood pressure response to dietary salt

**Figure 1:**
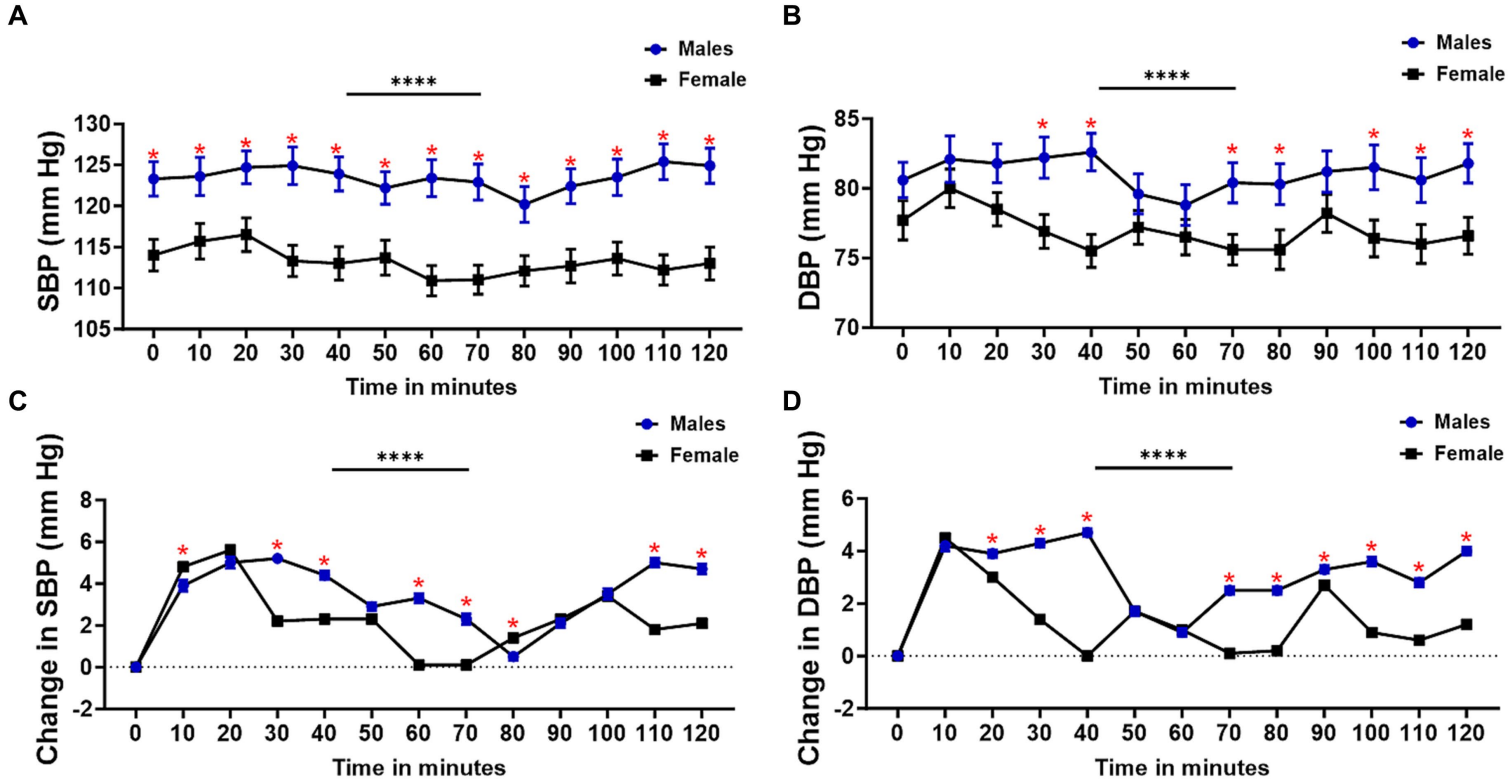
Sex differences in blood pressure response to dietary salt. **(A)** Systolic blood pressure (SBP), **(B)** diastolic blood pressure (DBP), **(C)** change in SBP, and **(D)** change in DBP from baseline (0 min) for males and females. Welch’s test was used to compare mean blood pressure between males and females **(A,B)**. Overall BP changes and changes at each interval were compared using a 2-way ANOVA with Šídák’s multiple comparisons test **(C,D)**. Asterisks indicate a time point with significant differences in blood pressure between males and females. *Interval with significant differences between blood pressure (*p* < 0.05); ****p < 0.0001.

Although the proportion of males and females did not differ between salt sensitive and salt resistant groups, we found significant sex differences in systolic and diastolic blood pressure response to dietary salt (Figure 1). Males had higher systolic blood pressure (SBP) than females at baseline, which was sustained throughout the 120 min after ingesting salt (Figure 1A). Diastolic blood pressure (DBP) for males and females were similar at baseline (time 0) but significant differences were noted at 30, 40, 70, 80, and 100–120 min with males recording higher DBP (Figure 1B). Overall, males had higher SBP (Figure 1C) and DBP (Figure 1D) increase compared to females.

**Figure 2:**
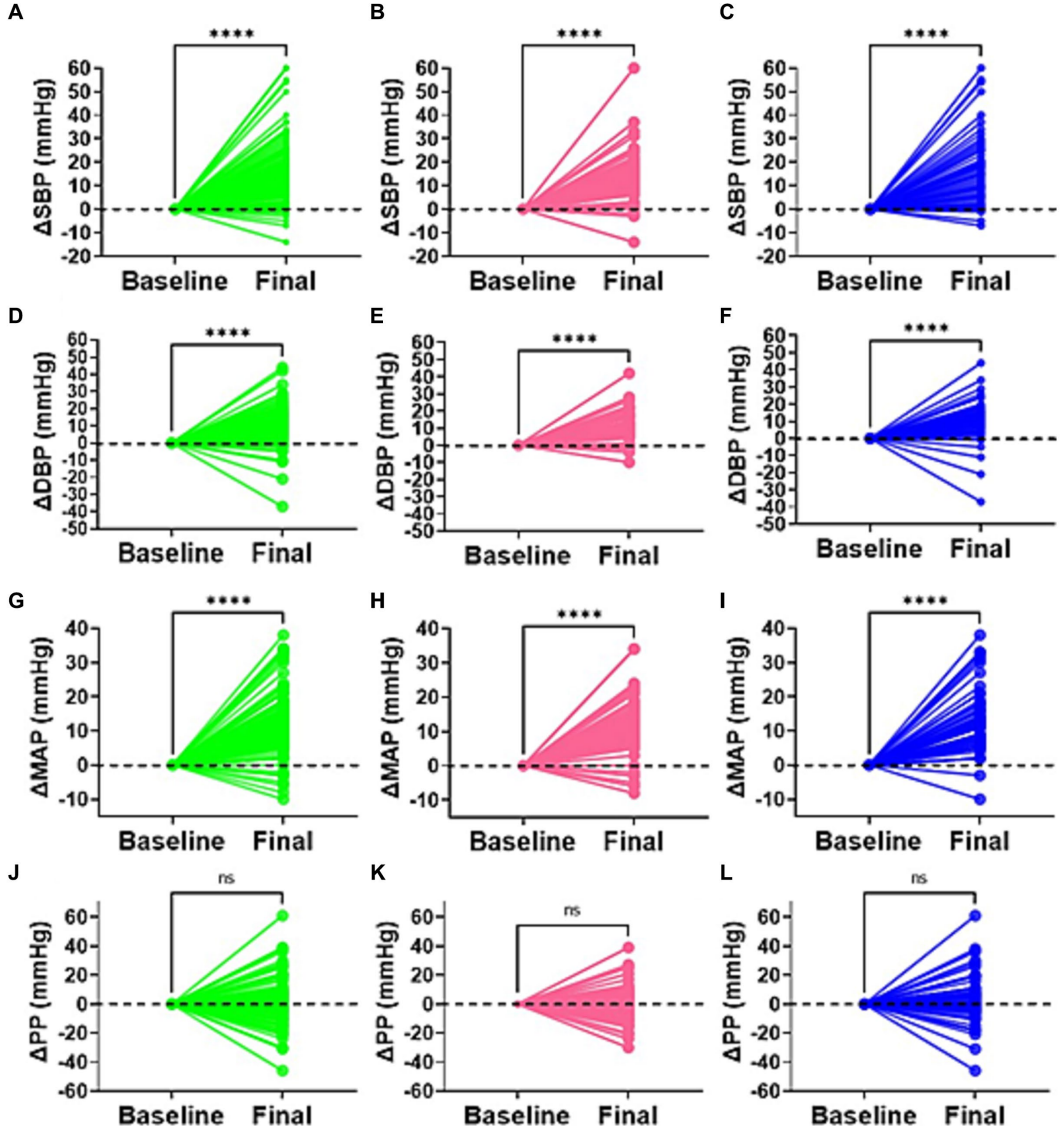
Delta difference in blood pressure. Changes in blood pressure between baseline and highest point (final) for all participants (green), females (pink), and males (blue) is indicated for **(A–C)** systolic blood pressure (SBP), **(D–F)** diastolic blood pressure (DBP), **(G–I)** mean arterial pressure (MAP), and **(J–L)** pulse pressure. ****p < 0.0001. ns, not statistically significant (*p* > 0.05).

To determine the blood pressure variability and degree of change in all participants, females and males, respectively, we compared the differences between the final and baseline blood pressure changes (Figure 2). We found that SBP, DBP and mean arterial pressure but not pulse pressure (PP), were significantly higher in all participants and for each sex following salt loading compared to baseline values. This suggested the presence of an immediate pressor response to oral salt.

### 3.3 Erythrocyte glycocalyx sensitivity to sodium and salt-sensitivity of blood pressure

**Figure 3:**
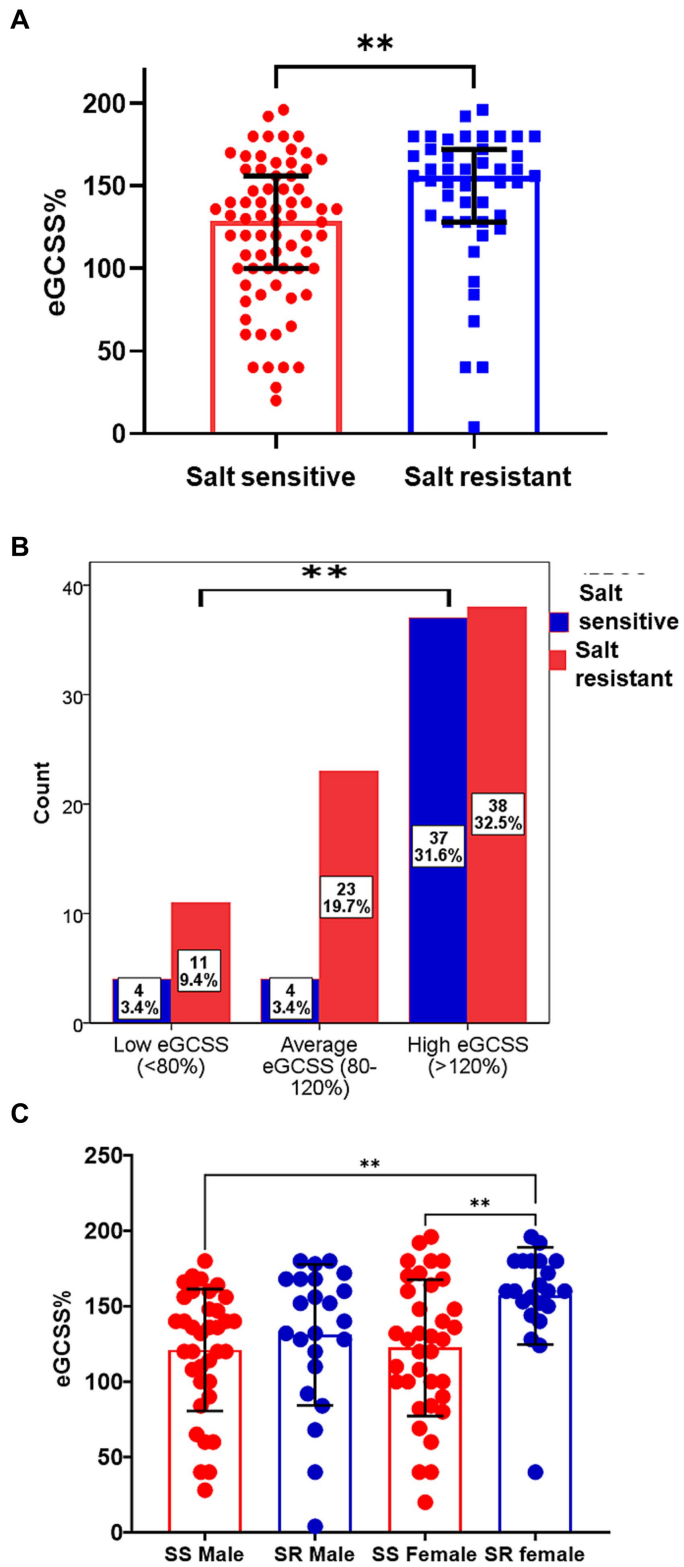
Erythrocyte glycocalyx sensitivity to sodium among salt sensitive and salt resistant groups. **(A)** The median (IQR) eGCSS among the salt sensitive [129% (100, 156,)] was lower compared to the salt resistant group[156% (128, 172, IQR)]. Mann–Whitney *U* test used. **(B)** SSBP status Stratification to eGCSS tertile Chi-square used. **(C)** Comparison of eGCSS between Salt sensitive (SS) and salt resistant (SR) males and females. eGCSS was higher in SR female compared to SS female and SS males; Brown-Forsythe and Welch ANOVA with Dunnett’s T3 multiple comparisons test. eGCSS, erythrocyte glycocalyx sensitivity to sodium; IQR, interquartile range; SSBP, salt sensitivity of blood pressure. **p < 0.01.

We compared eGCSS among salt sensitive and salt resistant participants and found that the salt resistant had a higher median (IQR) eGCSS [156% (128, 172, IQR)] when compared to the salt sensitive group [129% (100, 156)] (Figure 3A). We have previously compared the proportions of participants with SSBP to eGCSS tertile and found a significant relationship between the two characteristics (4). Compared to the salt resistant group, participants with SSBP were more in the low and average eGCSS tertile (Figure 3B) (4). We therefore compared eGCSS scores by sex between salt sensitive and salt resistant participants and found that eGCSS was higher in salt resistant females compared to salt sensitive females and males (Figure 3C).

**Figure 4:**
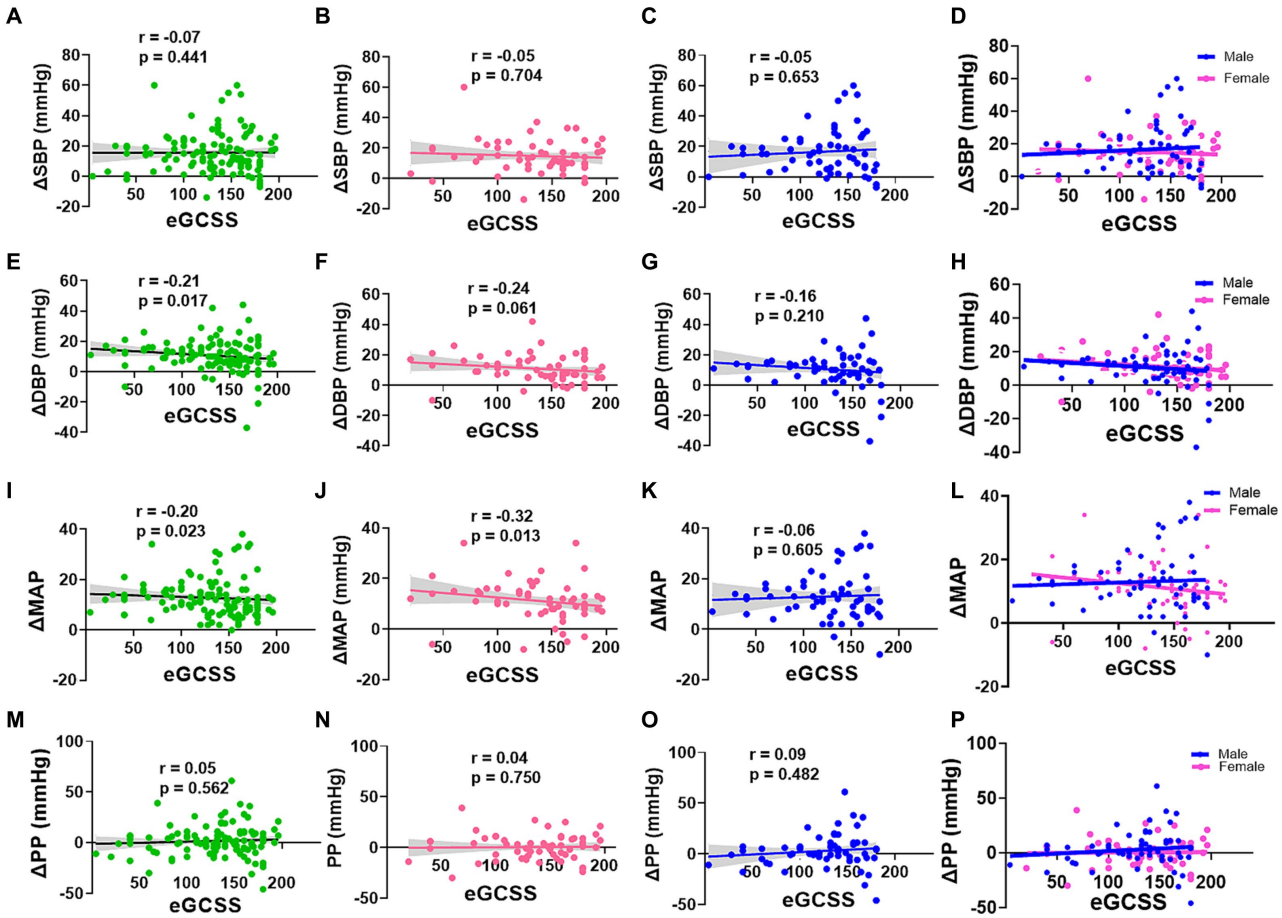
Correlation between Blood pressure change and Erythrocyte glycocalyx sensitivity to sodium by sex. SBP, DBP, MAP, and PP change (Δ) in all participants **(A,E,I,M)**, females **(B,F,J,N)**, males **(C,G,K,O)**, and males and females superinterposed **(D,H,L,P)** respectively, was performed using spearman’s correlation. SBP, systolic blood pressure; DBP, diastolic blood pressure (DBP); MAP, mean arterial pressure; PP, pulse pressure; eGCSS, erythrocyte glycocalyx sensitivity to sodium.

To further explore this relationship by sex, we then compared correlations between eGCSS and changes in blood pressure between baseline and highest change (delta) and found that SBP change correlated negatively with eGCSS in the whole group (Figure 4A), females (Figures 4B,D) and males (Figures 4C,D) but the relationship was not significant (*p* > 0.05). DBP change in the whole group (Figure 4E) correlated negatively with eGCSS and the correlation was significant (*r* = −0.21, *p* = 0.017) but not so when segregated by sex (Figures 4F–H). MAP correlated negatively with eGCSS in the whole group (Figure 4I) (*r* = −0.20, *p* = 0.023) and among the females (Figure 4J) (*r* = −0.32, *p* = 0.013) but not among the males (Figures 4K,L) (*r* = −0.06, *p* = 0.605) suggesting a specific sex-driven contribution. Pulse pressure (PP) correlated positively with eGCSS in the whole group (Figure 4M), in females (Figure 4N), and males (Figures 4O,P) but the relationship was not significant.

### 3.4 Sex differences in the association between erythrocyte glycocalyx sensitivity to sodium and salt-sensitivity of blood pressure

To determine the direction, magnitude and tertile of eGCSS associated with SSBP, we performed a logistic regression model and adjusted eGCSS for age, sex and body mass index (Table 1). We found that eGCSS correlated negatively with SSBP. In order to determine the contribution of each eGCSS category in predicting SSBP, we repeated the regression model using eGCSS tertile and found that participants with average eGCSS were 5.9 times more likely to have an SSBP compared to participants with high eGCSS. We repeated the model with eGCSS dichotomized and found that participants with low-to-average eGCSS were 4.4 times more likely to have SSBP compared to those with high eGCSS.

**Table 1:** Predictors of SSBP in logistic regression (all participants).

Variable	Odds ratio (OR) (95% CI)	*p*-value	Adjusted odds ratio AOR (95%CI)	*p*-value
Age, years	0.99 (0.97, 1.02)	0.844	0.99 (0.96, 1.02)	0.597
Sex				
Females	1		1	
Males	0.95 (0.45, 2.01)	0.907	0.82 (0.36, 1.83)	0.633
Body mass index (kg/m^2^)	1.00 (0.94, 1.06)	0.834	1.01 (0.95, 1.08)	0.629
*eGCSS scores	0.98 (0.97, 0.99)	**0.010**	0.98 (0.97, 0.99)	**0.008**
*eGCSS tertile (%)				
High (> 120)	1		1	
Low (< 80)	2.67 (0.78, 9.16)	0.117	2.89 (0.82, 10.14)	0.097
Average (80–120)	5.59 (1.76, 17.75)	**0.003**	5.99 (1.86, 19.28)	**0.003**
*eGCSS category (%)				
High (> 120)	1		1	
Low-to-average (≤120)	4.13 (1.69, 10.11)	**0.002**	4.46 (1.78, 11.13)	**0.001**

EGCSS, erythrocyte glycocalyx sensitivity to sodium. *Adjusted for age, sex and body mass index in the model. Bold values delineate *p* value <0.05.

To account for sex differences, we segregated the regression model in Table 1 by sex and found that the negative significant association between eGCSS and SSBP was driven by female sex (Table 2), given that the statistical significance was lost in the males (Table 3).

**Table 2:** Predictors of SSBP among females in logistic regression.

Variable	Odds ratio (OR) (95% CI)	*p*-value	Adjusted odds ratio AOR (95%CI)	*p*-value
Age, years	0.99 (0.95, 1.03)	0.786	0.98 (0.94, 1.02)	0.398
Body mass index (kg/m^2^)	1.04 (0.96, 1.13)	0.307	1.03 (0.93, 1.13)	0.508
*eGCSS	0.97 (0.95, 0.99)	**0.007**	0.97 (0.95, 0.99)	**0.008**
*eGCSS tertile (%)				
High (> 120)	1		1	
Low (< 80)	5.52 (0.59, 51.64)	0.134	5.23 (0.52, 52.17)	0.159
Average (80–120)	22.06 (0.57, 98.84)	0.970	23.06 (0.53, 102.96)	0.990
*eGCSS category (%)				
High (> 120)	1		1	
Low-to-average (≤120)	18.78 (2.27, 154.99)	**0.006**	21.09 (2.45, 181.65)	**0.006**

EGCSS, erythrocyte glycocalyx sensitivity to sodium. *Adjusted for age, and body mass index in the model. Bold values delineate *p* value <0.05.

**Table 3:** Predictors of SSBP among males in logistic regression.

Variable	Odds ratio (OR) (95% CI)	*p*-value	Adjusted odds ratio AOR (95%CI)	*p*-value
Age, years	1.00 (0.96, 1.03)	0.982	1.00 (0.96, 1.05)	0.748
Body mass index (kg/m^2^)	0.93 (0.84, 1.04)	0.237	0.93 (0.82, 5.64)	0.317
*eGCSS tertile	0.99 (0.98, 1.01)	0.380	0.99 (0.98, 1.00)	0.544
*eGCSS tertile (%)				
High (> 120)	1		1	
Low (< 80)	1.68 (0.36, 7.83)	0.506	1.39 (0.27, 6.96)	0.688
Average (80–120)	2.31 (0.61, 8.70)	0.214	2.06 (0.53, 7.96)	0.292
*eGCSS category (%)				
High (> 120)	1		1	
Low-to-average (≤120)	2.04 (0.67, 6.16)	0.204	1.78 (0.56, 5.64)	0.323

EGCSS, erythrocyte glycocalyx sensitivity to sodium. *Adjusted for age, and body mass index in the model.

## 4 Discussion

The goal of our study was to determine the sex differences in the relationship between eGCSS and SSBP and other variables in young normotensive black adults. There is currently no standard protocol for the diagnosis of SSBP and the current methods are still not feasible in clinical settings. We used an acute protocol to identify an immediate pressor response to an oral dose of NaCl to define SSBP. This is the first study known to us to demonstrate that eGCSS correlated negatively with SSBP in a sex-specific manner.

**Figure 5:**
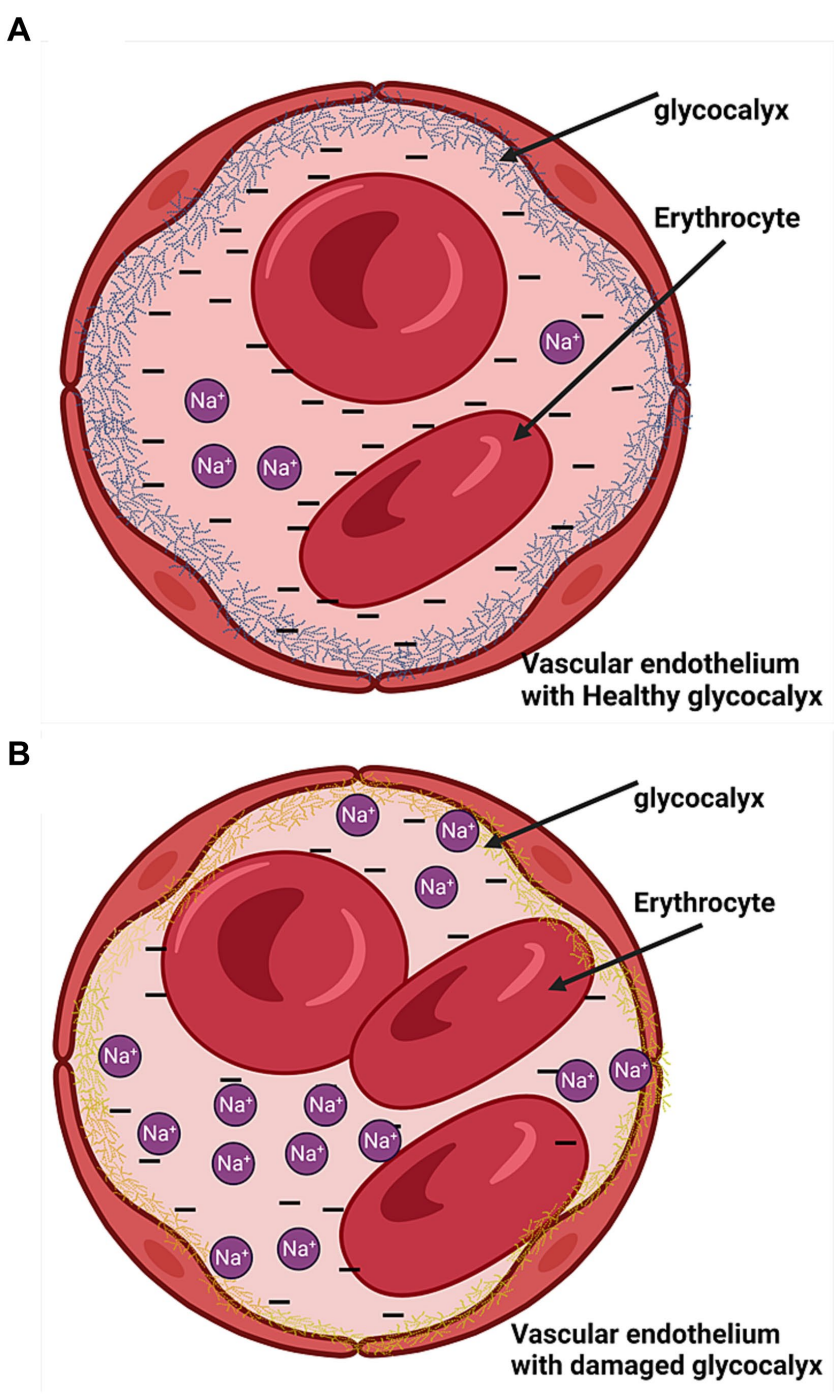
Endothelial cells with the glycocalyx. **(A)** Health glycocalyx. **(B)** Damaged glycocalyx (yellow).

The erythrocyte and vascular endothelial glycocalyces have negatively charged heparan sulfate residues that inhibit erosion on each other by repelling effects and selectively bind sodium (Figure 5A) such that in excess sodium intake, the glycocalyx’s buffering capacity is decreased resulting in erosion and damage to the glycocalyces (Figure 5B) (22, 23). We recently found that high salt intake correlates with a high eGCSS (6). The eGCSS is a reflection and marker of cell membrane damage in hypertension due to sodium retention characterized by a low-renin state as demonstrated recently by McNally et al. (17).

Chronic high salt intake is well recognized as a risk factor for future hypertension and mortality (1, 2), but the acute effects of salt and its relation to the status of the erythrocyte and endothelial glycocalyx are not well studied. A study by Dickinson et al. demonstrated that salt intake can have immediate effects on endothelial function assessed by brachial artery flow-mediated dilatation (FMD) (24). Compared to a low salt meal, consuming a high salt meal containing 65 mmol of Na^+^ suppressed FMD at 30 and 60 min postprandial without any significant differences in blood pressure and reactive hyperemia index. However, in a similar study by Wahab et al. (25) where participants consumed a high salt diet containing 4 g of salt equivalent to 68 mmol of Na^+^, they found that FMD increased along with plasma sodium, correlating with carotid pulsatility index. In both studies, they did not measure eGCSS in order to make comparisons with glycocalyx health. Our study also did not assess FMD or carotid pulsatility index to relate with endothelial function. However, evidence for the effect of salt on endothelial function is abundant (24, 26–30). Excess sodium acts on the endothelium and cells of the immune system resulting in oxidative stress, increased endothelial cell stiffness, damage to the endothelial glycocalyx and ultimately, endothelial dysfunction (31–34).

We found that eGCSS correlated negatively with SSBP, especially in individuals with average eGCSS but the mechanism is not known and is beyond the scope of this current study. It is conceivable that in individuals with high eGCSS, the damage to the glycocalyx and endothelium, which is associated with arterial stiffness and poor conduit artery elasticity may inhibit early detection of an acute response to salt. In contrast, in individuals with low-to-average eGCSS, arterial function and elasticity is preserved, and it can be detected early. Further follow-up studies are required to ascertain this relationship.

Although blood pressure was significantly higher in both males and females during the time course compared to baseline, systolic and diastolic blood pressure were consistently higher in males than in females. We found that blood pressure changes from baseline and several time points (SBP at 10, 30, 40, 60 70, 80, 110, and 120 and DBP at 20, 30, 40, and 70–120 min) were higher in males than in females. It is well known that males tend to have higher blood pressure compared to premenopausal females (9, 35, 36). While eGCSS was associated with SSBP, this relationship was abrogated by the male sex. Why the significant association was sustained in females remains to be explored and is beyond the scope of this study. However, emerging evidence now indicates that women in premenopausal age are more salt sensitive compared to men (37). Thus, women’s vascular function and response to salt may be physiologically deranged in SSBP compared to men. It is important to also emphasize that black individuals are more salt sensitive compared to white individuals and this difference may be determined by a lot of factors such as disparities in historical and contemporary socioeconomic status, genetic susceptibility, sodium handling by the kidney, the renin-angiotensin-aldosterone system and the autonomic nervous system’s response to salt, and environmental factors (38–42). However, our study was limited to black individuals and therefore could not determine racial disparities.

### 4.1 Clinical implications

#### 4.1.1 Clinical implications of high eGCSS

Most participants had high eGCSS. From what we know so far about eGCSS, it is a novel test that reflects endothelial and erythrocyte surface damage but its utility in clinical practice has not yet been established. Results from multiple studies found that high eGCSS was associated with low renin levels and high albumin-creatinine ratio in hypertension (17), correlated with high salt intake in a similar population from Zambia (6), and was associated with hypertension despite use of antihypertensive medication (43). One study also found that about one third of healthy individuals exhibit a high eGCSS (16) suggesting that eGCSS can be influenced by environmental factors such as dietary salt (44). An experimental study by Oberleithner et al. (22) demonstrated that sodium renders endothelial cells sticky to red blood cells suggesting a potential contribution to thrombotic events. The sex differences in correlates of eGCSS have not been studied and future studies are needed to determine mechanisms.

#### 4.1.2 Clinical implications of an acute SSBP and ABI

The bimodal pattern of sustained SBP and DBP changes during the 2-h time course in this study is interesting and akin to the broader 24-h ambulatory blood pressure changes that were described by Degaute et al. (45) three decades ago. However, the bimodal pattern of blood pressure change in our study was slightly different in men and women. In men, the highest SBP were recorded at 30- and 110-min and the highest DBP at 40- and 120-min post salt load. In women the highest SBP were recorded at 20 and 100-min while the DBP were highest at 10- and 90-min. This suggests that women respond earlier to the pressor effect of salt than men. Our data also demonstrates that men have a more severe increase in blood pressure with blood pressure elevations that do not return to baseline within 2 h compared to women. In women, SBP and DBP returned to baseline twice and thrice, respectively, during the 2-h time course. This may suggest that the stimulus responsible for the initial elevation in SBP and DBP in men does not dissipate quickly in men compared to women. However, more studies are required to validate this.

A tendency to have significant perturbations in blood pressure may contribute to acute or chronic blood–brain barrier disruptions depending on the magnitude and duration of the perturbation (46). Generally, it is normal for blood pressure to fluctuate during the day but significant and chronic perturbations arising from dietary salt may be associated with elevated 24-h ambulatory blood pressure, worsening of non-dipping blood pressure and increase the risk for hypertension (47). A randomized, double-blind crossover study by Tzemos et al. (48) found that 5-day acute salt loading impaired endothelial function, electric repolarization and left ventricular mechanical relaxation in young healthy normotensives. We used a one-dose salt load to monitor its impact on blood pressure in young normotensive black individuals who were seated for 2 h and whose blood pressure increase was sustained for the entire follow up period in both sexes. Our study suggests two aspects of clinical significance. First, with three routine meals per day customary for most Zambians that have similar or higher salt load, blood pressure increases are likely to be more significant in increasing the 24-h ambulatory blood pressure. With chronic high salt habits, this may likely increase the risk for the development of hypertension. The second implication is that if blood pressure is significantly raised in a sedentary sitting position, higher blood pressure perturbations are likely to occur with routine activity and consequentially increase vascular stress on blood vessels and hypertensive tendencies. However, these hypotheses need to be validated in future studies.

It was also interesting to find that 5.1% of this young population studied had evidence of peripheral artery disease or narrowing of peripheral arteries as evidenced from a higher ABI. Sub-analysis of this data (not shown) indicated that none of the participants with peripheral artery disease were living with HIV and the distribution by sex was comparable. However, the number of participants with abnormal ABI was not sufficient to draw any conclusions.

### 4.2 Strengths and limitations

To our knowledge, this is the first study to report sex dimorphism mediating the relationship between eGCSS and SSBP in a young population of normotensive black individuals. In the main prior study, we could not find a significant relationship between eGCSS and SSBP because we only included eGCSS tertiles in the multivariable analysis and adjusted for more variables that were not significant at univariable analysis (4). However, in this sub study, we used three models with fewer variable adjustments (adjusting only for variables that influence the outcome) to avoid overfitting the regression model. In the first instance, eGCSS was added to the logistic regression model as a continuous variable, then we repeated the analysis replacing the continuous variable eGCSS with eGCSS tertiles and lastly, we dichotomized eGCSS into high and low-to-average categories (Table 3).

The experimental nature of the study to determine SSBP is one of the strengths of this study. Although there are different protocols used to define SSBP with variations in blood pressure responses (49, 50), the choice of using an oral salt loading protocol to define SSBP is appropriate in the context of studies focused on the immediate effects of salt on blood pressure. However, acute salt loading protocols may underestimate the burden of SSBP. Protocols involving longer periods of salt loading are more accurate and have higher sensitivity in determining SSBP status. eGCSS is a potentially useful marker of sodium sensitivity to damage induced by dietary salt at the cellular level (17) but requires further validation studies to ascertain whether it can be used in clinical settings. For studies assessing sex differences within subcategories of eGCSS, this study may not have been adequately powered. To generalize and validate these findings, a study with a larger sample size is required. Another limitation is that there was no information collected regarding the stage of the menstrual cycle during the time the women participated in the study. Variations in blood pressure in women that reflects the stage of menstrual cycle has been reported in literature (51, 52).

Although eGCSS was associated with SSBP and driven by female sex, there are several markers of endothelial function and factors that influence eGCSS such as previous habitual intake of salt that were not measured in this study. This is a major limitation. It was going to be important to also report on additional factors that influence salt handling such as glomerular filtration rate, plasma levels of insulin, renin, aldosterone, and angiotensin II. These data were not available in this study and will be the focus of future studies. However, renal and kidney function tests were measured to exclude participants with any kidney or liver abnormality but have not been presented as they were not intended for statistical analyses prioli. It is possible that because we only included a younger population in this study, variances in both eGCSS scores and SSBP changes which are affected by age could not be elaborated. The level of physical activity may likely affect eGCSS and was not determined in this study. The findings from this study should therefore be interpreted in the context of the specific characteristics of this population. We are currently studying dietary salt effects in our laboratory, and we plan to have additional robust techniques to assess endothelial function from the effects of dietary salt in future. Finally, this was a cross sectional study and does not implicate causality.

## 5 Conclusion

The eGCSS correlated negatively with SSBP in females but not in males in this study and the immediate pressor response to dietary salt was significantly higher in males compared to females. The implication of our findings suggests that there could be a novel underlying mechanism linking eGCSS with acute salt loading. However, more studies are required to elucidate this link and robust markers of endothelial function are required to validate the role that eGCSS plays in SSBP.

## Data availability statement

The raw data supporting the conclusions of this article will be made available by the authors, without undue reservation.

## Ethics statement

The studies involving humans were approved by the University of Zambia Biomedical Research Ethics committee. The studies were conducted in accordance with the local legislation and institutional requirements. The participants provided their written informed consent to participate in this study.

## Author contributions

SM: Conceptualization, Data curation, Formal analysis, Funding acquisition, Investigation, Methodology, Project administration, Resources, Validation, Visualization, Writing – original draft, Writing – review & editing. BH: Data curation, Validation, Writing – review & editing. KP: Validation, Visualization, Writing – review & editing. AK: Formal analysis, Funding acquisition, Methodology, Validation, Visualization, Writing – original draft, Writing – review & editing.
