# Supplementary material for: Erythrocyte glycocalyx sensitivity to sodium is associated with salt sensitivity of blood pressure in women but not men
Source: Front Nutr. 2024 Mar 8;11:1334853. doi: 10.3389/fnut.2024.1334853 (PMC10957757; doi:10.3389/fnut.2024.1334853)
Supplement: Supplementary file 1 [file Table_1.pdf]

Supplementary Material

Table 1: Distribution of characteristics by SSBP status

**Variable**	**SSBP**		**p-value**	***All***
	**Salt sensitive** (MAP≥10 mmHg), **n (%) =72 (61.5%)**	**Salt resistant** (MAP< 10 mmHg) **n (%) =45 (38.5%)**		
**Age**, *median years (IQR)*	27 (22, 45)	30 (23, 47)	0.680	29 (22-45)
**Sex**				
Male	36 (50.0)	23 (51.1)	0.907	59 (50.40)
Female	36 (50.0)	22 (48.9)		58 (49.6)
**Marital status**				
Married	21 (29.2)	20 (44.4)	0.151	41 (35.0)
Single	42 (58.3)	23 (5.1)		65 (55.6)
Divorced or separated	4 (5.6)	2 (4.4)		6 (5.1)
Widowed	5 (6.9)	0 (0.0)		5 (4.3)
**Employment status**				
Employed	13 (18.1)	9 (20.0)		22 (18.8)
Unemployed	54 (75.0)	33 (73.3)		87 (74.4)
Retired	5 (6.9)	3 (6.7)		8 (6.8)
**Body mass index**, kg/m^2	23.4 (21.0, 26.3)	23.0 (20.3, 28.2)	0.93	23.0 (20.7-27.4)
**Ankle brachial index**	1.01 (1.00, 1.10)	1.03 (1.00, 1.10)	0.60	1.00 (1.00, 1.10)
**Ankle brachial index category**				
Normal	68 (94.4)	43 (95.6)	0.791	111 (94.9)
Peripheral artery disease	4 (5.6)	2 (4.4)		6 (5.1)
**Fasting blood sugar**, *n=104, mmol/l*	4.7 (4.3, 5.2)	4.7 (4.2, 5.0)	0.84	4.7 (4.2, 5.1)
**eGCSS (%)**	131 (92, 159)	156 (128, 172)	**0.002**	140 (104, 164)
**eGCSS category (%)**				
High (> 120)	34 (47.2)	8 (17.8)	**0.001**	42 (35.9)
Low-to-average (≤120)	38 (52.8)	37 (82.2)		75 (64.1)
**eGCSS tertile (%)**				
Low (< 80)	11 (15.3)	4 (8.9)	**0.004**	15 (12.8)
Average (80–120)	23 (31.9)	4 (8.9)		27 (23.1)
High (> 120)	38 (52.8)	37 (82.2)		75 (64.1)
				140 (104, 164)
**Red blood cell count** *×10^{12}/L , n=110*	4.61 (3.96, 5.15)	4.74 (3.71, 4.95)	0.91	4.61 (3.91–5.09)
HIV status				
Positive	13 (18.1)	8 (17.8)	0.97	21
Negative	59 (81.9)	37 (82.2)		96
